# Influence of Varying Concentrations of Epoxy, Rice Husk, Al_2_O_3_, and Fe_2_O_3_ on the Properties of Brake Friction Materials Prepared Using Hand Layup Method

**DOI:** 10.3390/polym15122597

**Published:** 2023-06-07

**Authors:** Agustinus Purna Irawan, Deni Fajar Fitriyana, Januar Parlaungan Siregar, Tezara Cionita, Paula Tjatoerwidya Anggarina, Didi Widya Utama, Teuku Rihayat, Rusiyanto Rusiyanto, Saeful Dimyati, Muhammad Bustanul Aripin, Rifky Ismail, Athanasius Priharyoto Bayuseno, Gregorius Dimas Baskara, Muhammad Khafidh, Finny Pratama Putera, Rahmadi Yotenka

**Affiliations:** 1 Faculty of Engineering, Universitas Tarumanagara, Jakarta 11480, Indonesia; didiu@ft.untar.ac.id; 2 Department of Mechanical Engineering, Universitas Negeri Semarang, Kampus Sekaran, Gunungpati, Semarang 50229, Indonesia; me_rusiyanto@mail.unnes.ac.id (R.R.); saefuldhem@students.unnes.ac.id (S.D.); muhammadbustanularipin@students.unnes.ac.id (M.B.A.); 3 Faculty of Mechanical & Automotive Engineering Technology, Universiti Malaysia Pahang, Pekan 26600, Malaysia; januar@ump.edu.my; 4 Faculty of Engineering and Quantity Surveying, INTI International University, Nilai 71800, Malaysia; tezara.cionita@newinti.edu.my; 5 Faculty of Economics and Business, Universitas Tarumanagara, Jakarta 11480, Indonesia; paula@fe.untar.ac.id; 6 Department of Chemical Engineering, Politeknik Negeri Lhokseumawe, Lhokseumawe 24301, Indonesia; teukurihayat@yahoo.com; 7 Department of Mechanical Engineering, Faculty of Engineering, Diponegoro University, Semarang 50275, Indonesia; r.ismail.undip@gmail.com (R.I.); apbayuseno@gmail.com (A.P.B.); 8 School of Electrical Engineering and Informatics, Institut Teknologi Bandung, Bandung 40132, Indonesia; 13519190@std.stei.itb.ac.id; 9 Department of Mechanical Engineering, Universitas Islam Indonesia, Sleman, Yogyakarta 55584, Indonesia; khafidh@uii.ac.id (M.K.); 195250101@uii.ac.id (F.P.P.); 10 Department of Statistics, Universitas Islam Indonesia, Sleman, Yogyakarta 55584, Indonesia; 156111305@uii.ac.id

**Keywords:** brake friction materials, composites, rice husk, Al_2_O_3_, Fe_2_O_3_, sustainable development goals (SDGs)

## Abstract

Brake friction materials (BFMs) have a critical role in ensuring the safety as well as the reliability of automotive braking systems. However, traditional BFMs, typically made from asbestos, are associated with environmental and health concerns. Therefore, this results in a growing interest in developing alternative BFMs that are eco-friendly, sustainable, and cost-effective. This study investigates the effect of varying concentrations of epoxy, rice husk, alumina (Al_2_O_3_), and iron oxide (Fe_2_O_3_) on the mechanical and thermal properties of BFMs prepared using the hand layup method. In this study, the rice husk, Al_2_O_3_, and Fe_2_O_3_ were filtered through a 200-mesh sieve. Note that the BFMs were fabricated using different combinations and concentrations of the materials. Their mechanical properties, such as density, hardness, flexural strength, wear resistance, and thermal properties, were investigated. The results suggest that the concentrations of the ingredients significantly influence the mechanical and thermal properties of the BFMs. A specimen made from epoxy, rice husk, Al_2_O_3_, and Fe_2_O_3_ with concentrations of 50 wt.%, 20 wt.%, 15 wt.%, and 15 wt.%, respectively, produced the best properties for BFMs. On the other hand, the density, hardness, flexural strength, flexural modulus, and wear rate values of this specimen were 1.23 g/cm^3^, 81.2 Vickers (HV), 57.24 MPa, 4.08 GPa, and 8.665 × 10^−7^ mm^2^/kg. In addition, this specimen had better thermal properties than the other specimens. These findings provide valuable insights into developing eco-friendly and sustainable BFMs with suitable performance for automotive applications.

## 1. Introduction

The research and development of BFMs for automotive applications continues to grow, along with restrictions on the use of asbestos due to this substance’s carcinogenic and toxic properties [1,2,3,4]. The friction material of brakes is a composite used to stop and manage the vehicle’s speed by pressing against the rotating disc [5]. Note that the BFMs are worn during this process. The amount of wear is primarily determined by the kind of friction material used, the pressure applied to the pads, the operation temperature of the friction material, the friction material contact area, the friction material finish, the rate of heat removal, the ability to operate under different atmospheric conditions, and the resistance to fading [6]. In addition, the BFM is a consumable component that must be periodically replaced [7,8,9]. Consequently, the demand for BFMs is growing along with the number of motorcycles. The number of motorcycles in Indonesia will reach 126,350,426 units by the end of 2022 [10]. This statistical datum can be utilized as an illustration of the high demand for BFMs in the future.

Developing non-asbestos BFMs is crucial for various reasons, including health and environmental concerns, regulatory compliance, and performance standards [11,12]. Utilizing alternative materials, manufacturers can create BFMs that are safe, effective, and environmentally friendly. For example, the friction material in brake pads consists of binders, reinforcement, fillers, and abrasives. Each component is critical in creating the necessary friction and heat to stop a vehicle safely and reliably [13]. Here, binders are the substances that keep the friction material’s other ingredients together.

These are typically synthetic resins, such as phenolic and epoxy resins, that can withstand high temperatures and have excellent adherence to the other parts. The binder is an important part of the friction material as it keeps everything together and keeps up with the high temperatures and pressures involved. The friction material is reinforced with other materials to make it stronger and last longer. Wear and high temperatures are not a problem for these materials, which can be made from natural or synthetic fibers. Hence, the BFM’s durability is increased, and the service life is prolonged by employing reinforcement compounds that enable the friction material to survive the repeated stress of braking. The BFM’s properties can be modified by adding or removing fillers from the friction compound. Graphite, ceramic, and metal powders are all examples of common fillers. Subsequently, these improve the friction material’s uniformity and lower noise and vibration when braking. In order to improve the friction coefficient, or the amount of force needed to stop the vehicle, abrasives are added to the friction material. Metal oxides and silicates are two abrasives that can generate a rough surface on the brake rotor that then grips the rotor and slows the vehicle. The stopping power, noise level, and wear characteristics of a BFM can be affected by the type and amount of abrasive employed [4,14,15,16].

In the past decade, natural fibers have been investigated as potential sustainable components for automotive BFM reinforcement [17]. Numerous plant products, such as coconut shell waste, hemp, bamboo, palm kernel, kenaf, sawdust, sugar cane, sugarcane bagasse ash, coconut shell, Nile rose, wheat powder, rice husk powder, banana fiber, have been studied [18,19,20,21,22,23,24,25,26,27]. The utilization of rice husk as a sustainable and eco-friendly BFM is gaining popularity for its abundance, low cost, and unique attributes [28]. Although rice husks are currently utilized only in specialized applications such as zootechnics and horticulture, they can be utilized in other applications, such as the production of BFMs [18].

Therefore, rice husk has been exploited exaggeratedly in producing polymer matrix composites [29]. In contrast to other natural fibers, rice husk contains a significant amount of amorphous silica, potentially affecting brake pads’ tribological properties. Thus, utilizing rice husks to produce BFMs has the potential to produce a more eco-friendly and long-lasting alternative to conventional BFMs (asbestos) [18,29,30,31,32,33,34]. In addition to rice husk, various other ceramic materials, either fibers or particles, have been incorporated into the matrix to produce BFMs [35,36,37].

Despite expensive production costs and brittleness having limited their scope of application, ceramic materials have been utilized in special applications, such as high working temperatures, extremely acidic situations, and conditions requiring high wear resistance, such as BFMs [36]. Ceramics such as aluminum [36,37,38,39] and ferric [40,41] oxide have recently been developed to replace asbestos-based automotive friction materials. In general, incorporating Al_2_O_3_ in fiber and particle form into BFMs at a certain concentration can significantly reduce wear loss and increase wear resistance. This occurs because specimens of BFM containing Al_2_O_3_ have a lower maximum temperature during braking than specimens without Al_2_O_3_. Consequently, the reduced maximum temperature during braking results in less mass loss and increased wear resistance [36,37,38,39]. In addition, BFMs with higher Fe_2_O_3_ content have higher strength. Hence, the friction traces left on the surface are shallower [40,41]. However, an excessive amount of Fe_2_O_3_ (>15%) can cause the composite to peel, especially when working at high speeds [40]. Research by Xu et al. indicates that an excessive content of iron powder generates too much Fe_2_O_3_, causing the friction film to peel off easily, particularly at high speeds. According to the study, alloying and granulating carbonyl iron powder has a beneficial impact on the strengthening of the copper matrix. Increased carbonyl iron content can facilitate the formation of a friction film and enhance its thickness, hardness, and tensile strength. However, when the Fe content is greater than 10%, friction film formation is accelerated, and cracks between the thicker film and the softening matrix can form easily. This results in significant wear rates and a fluctuating friction coefficient [41].

Rice husk, Al_2_O_3_, and Fe_2_O_3_ have been extensively explored as fillers, abrasives, and reinforcements in manufacturing BFMs. However, to our knowledge, using rice husk, Al_2_O_3_, and Fe_2_O_3_ together to manufacture BFMs with an epoxy resin as a binder has not been widely explored. Therefore, the optimal composition of rice husk, Al_2_O_3_, Fe_2_O_3_, and epoxy to obtain the desired performance of the brake pad composite have not yet been determined. Furthermore, the purpose of this study is to assess the effect of incorporating rice husk, Al_2_O_3_, and Fe_2_O_3_ (with a particle size of 200 mesh each) into epoxy resin on the properties of BFMs, including wear resistance, thermal stability, hardness, density, morphology, and flexural strength. The use of rice husk as a filler in brake pad applications contributes to the achievement of sustainable development goals (SDGs) in addition to promoting environmental sustainability.

## 2. Materials and Methods

### 2.1. Materials

The materials used in this study were epoxy resin, hardener, Al_2_O_3_, Fe_2_O_3_, and rice husk. Al_2_O_3_ and Fe_2_O_3_ were obtained from PT Merck Tbk, Jakarta, Indonesia, with a particle size of 200 mesh. Table 1 lists the properties of Al_2_O_3_ and Fe_2_O_3_. The rice husk in this study was retrieved from rice processing in Gunungpati, Semarang City, Indonesia. Note that the rice husks were crushed with the assistance of a crusher machine, and the crushed rice husk was subjected to a 200-mesh sieving process to obtain rice husk powder. The rice husk powder was dried at 80 °C for 24 h in an oven.

The epoxy and hardeners used in this study are epoxy EPR-174 and hardener EPH-555, obtained from PT Justus Kimia Raya, Indonesia, with properties revealed in Table 2.

### 2.2. Specimen Fabrications

In this study, the fabrication of BFM specimens refers to the method applied in previous research. Different concentrations of epoxy resin, Al_2_O_3_, Fe_2_O_3_, and rice husks were employed to fabricate BFM specimens (Table 3) [47]. The epoxy-to-hardener ratio in this study was 3:1, stirring for 7 min to ensure a uniform mixture. Subsequently, rice husk, Al_2_O_3_, and Fe_2_O_3_ were added according to a predetermined composition and stirred for 15 min.

Consequently, the resulting mixture was poured into the prepared mold and leveled according to the height of the mold with a roller or brush. Before the mold was applied, a cleaning process was conducted to ensure that the mold was clean from dust and dirt. Correspondingly, a brush was used to apply the wax evenly over the entire mold surface. This was performed to ensure that the composite was easy to remove and did not stick to the mold. The formed composite was then dried for 24 h at room temperature before testing. After the composite was removed from the mold, the specimen was cut with the assistance of a laser cutting machine according to the American Society for Testing and Materials (ASTM) standard for each relevant test.

### 2.3. Testing and Characterizations

The composite material formed was tested to determine its physical properties, mechanical strength, wear resistance, and thermal resistance. Meanwhile, density testing was performed to establish the density of the BFM specimens. An electronic density meter (DME 220 series) from Vibra Canada Inc. (Mississauga, ON, USA) was used to conduct density testing following ASTM 792-08 [48,49].

Subsequently, flexural testing was conducted to obtain the flexural modulus and flexural strength values using the three-point bending test method according to ASTM D790 standards via HT-2402 Computer Servo Control Material Testing Machines from Hung Ta Instrument Co., Ltd., Sammutprakarn, Thailand. The specimen employed for testing was a rectangularly shaped sample with 127 mm × 12.7 mm × 3 mm dimensions. The crosshead speed applied in the flexural test was 2 mm/min at room temperature [48,49].

Hardness testing is a mechanical testing method that aims to determine a material’s resistance when loaded [50]. The test object experiences deformation when a certain loading force is applied to the test object.

The hardness testing used the HV method with a Microhardness Tester Fm-800 from Future-Tech Corp., Kanagawa, Japan.

The wear test utilized the Ogoshi method, where the test object receives the friction load from the revolving disc. This frictional loading results in contact between the surfaces of the test object. This test employs the Ogoshi High-Speed Universal Wear Testing Machine (type OAT-U) from Tokyo testing machine Mfg. Co., Ltd., Aichi, Japan. The disc width (B), disc radius (r), and disc track length during rotation (L) employed in this study were 3 mm, 13.12 mm, and 66.6 m, respectively. The wear tests were conducted with a test load (F) of 2.12 kg and a wear distance of 15 m rotating at a speed of 1430 rpm [51]. The density, flexural, hardness, and wear tests were repeated thrice. Then, calculations were performed to determine the average value.

Differential scanning calorimetric (DSC) analyses for BFMs were performed employing a DSC 8000 DSC from PerkinElmer, Inc., Hopkinton, MA, USA. The nitrogen flow rate was 20 mL/min and the heating rate was 10 °C/min for the test, which was conducted up to 350 °C above room temperature. DSC testing is a thermal analysis technique that measures the energy absorbed or emitted by a sample as a function of time or temperature and is carried out per ASTM D3418 standard [52].

To study the thermal stability of the BFMs, a STA7200 thermogravimetric analyzer from Hitachi High-Tech Science Corporation, Tokyo, Japan, was utilized. A powdered 10 mg sample was put in an Al_2_O_3_ crucible and kept in the furnace. The analysis was completed in a controlled environment where 20 mL/min of nitrogen gas flowed. Note that the temperature change rate was kept at 10 °C/min while the experiment was performed from 30 °C to 700 °C. Thermal gravimetry analysis (TGA) testing was performed to obtain quantitative measurements of mass changes in the materials associated with the transitions of dehydration, decomposition, and oxidation of the samples with time and temperature functions, which were implemented according to ASTM D6370 standards [52].

Scanning electron microscope (SEM) testing in this study utilized a Hitachi TM3030 Plus machine from Hitachi High-Tech Science Corporation, Tokyo, Japan. This method was utilized to examine the fracture and fracture surface morphological behavior of flexural specimens. Each sample was coated with a thin palladium layer to prevent a surface charge prior to being mounted on a tool holder using electrically conductive carbon adhesive tape. Consequently, the sample was analyzed using a microscope with a specific magnification and 10 kV of accelerating voltage [47].

## 3. Results and Discussion

**Figure 1:**
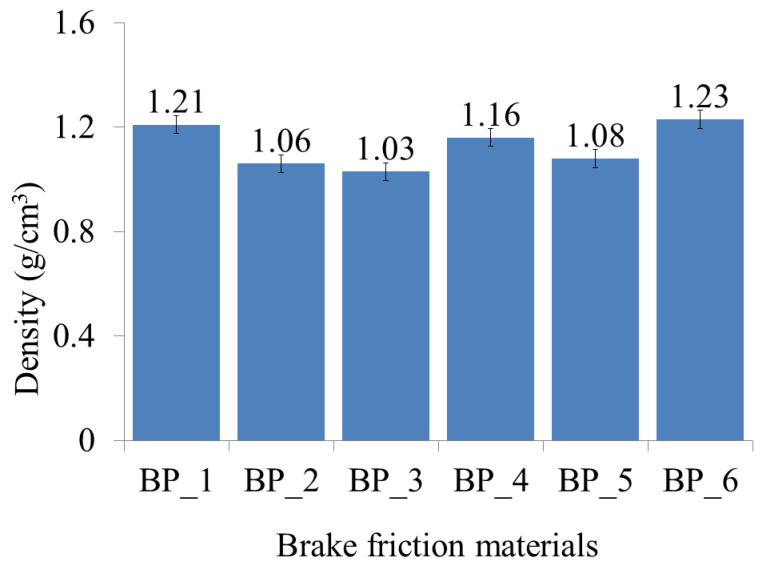
The densities of the brake friction material specimens.

The density of the BFMs with various compositions of the constituent materials are illustrated in Figure 1. The results of this study indicate that the addition of rice husk, Al_2_O_3,_ and Fe_2_O_3_ greatly affects the density of the resulting BFMs. The BP_6 specimen composed of epoxy, rice husk, Al_2_O_3_, and Fe_2_O_3_ with percentages of 50%, 20%, 15%, and 15%, respectively, produced a higher density than the other specimens, 1.23 gr/cm^3^. The results of this study indicate that the BP_3 specimen, which was composed of epoxy, rice husk, Al_2_O_3_, and Fe_2_O_3_ with percentages of 50%, 5%, 22.5%, and 22.5%, respectively, produced the lowest density, which was 1.03 gr/cm^3^. Meanwhile, the density of the epoxy resin (specimen BP_1) was 1.21 gr/cm^3^.

Abutu et al. (2019) proved that the density of commercial BFMs ranges from 1.010 to 2.060 g/cm^3^ [53]. Based on the research results, all BFM specimens produced in this study met the minimum density requirements. However, only the BP_6 specimen has a higher density compared to the density of the epoxy resin or BP_1 specimen. Meanwhile, the BP_2, BP_3, BP_4, and BP_5 specimens have lower densities than the density of epoxy resin. This demonstrates that applying the ingredient composition to the BP_6 specimen can produce composites with fewer voids. In addition, the composition of the BP_6 specimen provides composites with strong interfacial bonds between the rice husk, Al_2_O_3_, Fe_2_O_3_, and epoxy. This is the reason for the higher density of BP_6 specimens than the epoxy resin (BP_1) and other composite specimens. The content of Al_2_O_3_ and Fe_2_O_3_ in the BP_6 specimen was lower than in the BP_2, BP_3, BP_4, and BP_5 specimens.

The BP_2, BP_3, BP_4, and BP_5 specimens contained Al_2_O_3_, and Fe_2_O_3_ in concentrations (wt.%) of 25, 22.5, 20, and 17.5, respectively. Meanwhile, the BP_6 specimen contained 15 wt.% Al_2_O_3_ and 15 wt.% Fe_2_O_3_. Al_2_O_3_ and Fe_2_O_3_ are materials that have a high density. The densities of Al_2_O_3_ and Fe_2_O_3_ are 3.94 g/cm^3^ and 5.25 g/cm^3^, respectively (Table 1). Research conducted by Ergün demonstrated that low-density reinforcing material would be easier to distribute uniformly in the matrix during composite fabrication. As it becomes more difficult to acquire homogeneous mixtures when reinforcing materials with a high density, the remaining particle agglomerates form stress concentrations in the matrix, which can act as notches [54]. In addition, the presence of agglomeration in the composite can cause an increase in the number of voids, a decrease in interfacial bonding, and a decrease in density [54,55].

Rice husk has a high amorphous silica content, about 95% [18]. Thus, increasing the amount of rice husk employed in this study significantly increased the amorphous silica content in the BFM specimens. Other than that, rice husk can increase the density of BFMs due to its high content of amorphous silica, acting as a filler material in the composite mixture. When rice husk is added to BFMs, the amorphous silica particles can fill the voids between other particles in the mixture more efficiently, resulting in a higher packing density and ultimately leading to a higher overall density of the BFMs. In addition to amorphous silica, rice husk also contains other organic components, such as lignin, cellulose, and hemicellulose, that can contribute to the increased density of the brake pad material. These components can provide additional strength and stiffness to the composite mixture, making it more compact and dense. Moreover, rice husk absorbs less water than other biomass or natural fillers, including textile waste, bagasse ash, kenaf fiber, wood fiber, bamboo fiber, and wheat husk [56,57,58]. This is due to the fact that rice husk is more hydrophobic than other materials [56,59]. The lower the water absorption, the greater the composite’s density [60].

The other research mentioned that a composite with a high silica content can increase density. This occurs due to the increased adhesion between the filler and matrix, which results in a more compact structure and increased density [61,62]. Jiang et al.’s research demonstrated that a 20% silica concentration with a particle size of 5 μm produces a PTFE/SiO_2_ composite with a higher density than other specimens. Nonetheless, the density of the composite decreases if the silica content exceeds 20%, given that agglomerates are able to reduce adhesion between the filler and matrix [61].

The findings in this study align with research conducted by Dahham et al. [63]. Their research results suggest that increasing rice husk content produced composites with higher densities. In the research, the density of composites made with fine rice husk was greater than that of composites made with coarse rice husk. This is related to the fact that fine rice husks are more easily incorporated into the SMR L matrix, thereby increasing the rigidity and stiffness of the composites [63]. Alternatively, Fuad et al. also studied the effect of incorporating rice husk as a filler on the density of composites. In a polypropylene matrix, they utilize rice husks with a 0 to 40% percentage. A composite containing 40% rice husk had a greater density than other specimens [64].

This research suggests that adding Al_2_O_3_ and Fe_2_O_3_ powders at volume fractions of 15% each is an effective composition for increasing BFM density. This is due to the powder being able to fill the pores in the composite material, resulting in a denser, more compact structure. Compared to other specimens, the density of the BP_6 specimen containing Al_2_O_3_ and Fe_2_O_3_ powders with volume fractions of 15% each increased significantly. Nonetheless, when the volume fraction of these powders exceed 15%, the composite material’s density decreases. Furthermore, considering the high volume fractions, the composite material mixture can become increasingly inhomogeneous and agglomerate, increasing porosity and decreasing density [40,65].

In addition, the higher the concentrations of Al_2_O_3_ and Fe_2_O_3_ employed, the more difficult it is to achieve a homogeneous mixture during the mixing process. Al_2_O_3_ and Fe_2_O_3_ have a higher density than the other components of the mixture (epoxy and rice husk). Consequently, they are able to settle to the bottom of the mixing vessel, resulting in uneven distribution and inhomogeneity. Specimens with a high degree of uneven distribution and inhomogeneity may have increased porosity and decreased density. This is because when the components of a mixture are not evenly dispersed, there are voids or spaces between the particles, which can increase the overall porosity of the material. In addition, the uneven distribution can produce areas of material with varying densities, which can also contribute to a reduction in the overall density of the material. This is the reason why BP_2, BP_3, BP_4, and BP_5 have lower density than BP_1 (epoxy 100 wt.%).

The findings in this study are the same as those of a study conducted by Talib et al. [40] and Xu et al. [41]. The volume fraction effect (vol.%) of Fe_2_O_3_ on density in BFMs was studied by Talib et al. The results of their experiment indicate that the use of Fe_2_O_3_ of 7.5 vol.%, 15 vol.%, and 22.5 vol.% produced BFMs with densities of 2.01 g/cm^3^, 2.39 g/cm^3^, and 2.14 g/cm^3^, respectively [40]. Note that an excessive amount of Fe_2_O_3_ (>15 vol.%) can cause the composite to peel, especially when working at high speeds [40]. Research conducted by Xu et al. suggests that an excessive iron powder generates too much Fe_2_O_3_, which causes the friction film to peel off easily, particularly at high speeds [41].

The highest density in this study was discovered in the BP_6 specimen, 1.23 g/cm^3^. The density of the BP_6 specimen was higher than those of previous studies, 1.10 g/cm^3^ [66] and 1.073 g/cm^3^ [53]. However, the density of the BP_6 specimen was lower than the densities produced in other studies, equal to 2.29–2.59 g/cm^3^ [67] and 1.59–1.89 g/cm^3^ [68].

**Figure 2:**
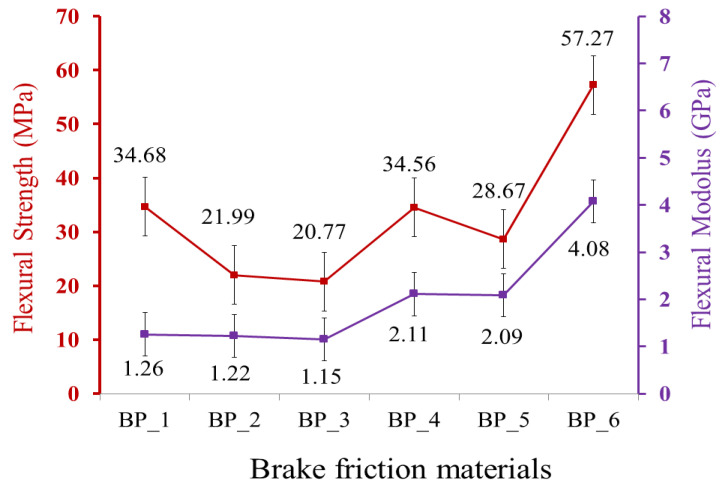
Flexural strength and flexural modulus of brake friction materials.

The flexural properties of the BFMs with various compositions of the constituent materials are displayed in Figure 2. The results of this study indicate that the addition of rice husk, Al_2_O_3,_ and Fe_2_O_3_ greatly affects the flexural properties of the resulting BFMs. The red and purple lines in Figure 2 show flexural strength and flexural modulus, respectively. Here, the highest flexural strength and modulus were produced in the BP_6 specimen. The flexural strength and modulus of the BP_6 specimen were 57.27 MPa and 4.08 GPa, respectively. The results of this study indicate that the BP_3 specimen produced the lowest flexural strength and flexural modulus, which were 20.77 MPa and 1.15 GPa. Meanwhile, the flexural strength and flexural modulus of the epoxy resin (BP_1 specimen) were 34.68 MPa and 1.26 GPa. Based on the research results, only the BP_6 specimen had higher flexural strength and flexural modulus than the flexural strength and flexural modulus of the BP_1 specimen.

Meanwhile, the specimens BP_2, BP_3, BP_4, and BP_5 have lower flexural strength than the epoxy resin flexural strength. Increasing concentrations of Al_2_O_3_ and Fe_2_O_3_ in the BP_2, BP_3, BP_4, and BP_5 specimens may result in bonding defects with the matrix. This reduced interfacial strength and increased concentration at the interface. As a result, load transfer efficiency and flexural strength were drastically reduced [69]. Consequently, the results of this study indicate that the BP_6 specimen has higher flexural properties than the specimens BP_1, BP_2, BP_3, BP_4, and BP_5.

According to the findings of this study, flexural strength and flexural modulus exhibit the same trend. An increase in the composite’s flexural strength is followed by an increase in its flexural modulus and vice versa. This study’s findings are consistent with the research conducted by Hossain et al. Their findings demonstrate that the flexural strength of carbon fiber–epoxy composites follows the same trends as the flexural modulus [70]. Similar results were found in studies by Islam et al. [71] and Fitriyana et al. [72]. Flexural modulus measures a material’s resistance to bending deformation. Meanwhile, flexural strength measures the maximum stress a material can withstand when bent [73].

In general, materials with higher flexural modulus tend to have higher flexural strength, as the material can better resist deformation and stress [73]. The results of Rahman et al.’s research indicate that increased filler loading in the polypropylene matrix increases flexural strength and modulus. This occurs due to the increased interface interaction between the fiber and the matrix, increasing the effective surface area available for contact with the matrix and the potential for load transfer between the matrix and reinforcing fiber. Consequently, flexural strength and modulus increased. Their research results also note that an increase in flexural modulus followed the increase in the flexural strength of the composite [74]. The findings of Li et al. also demonstrate the same characteristic [75].

**Figure 3:**
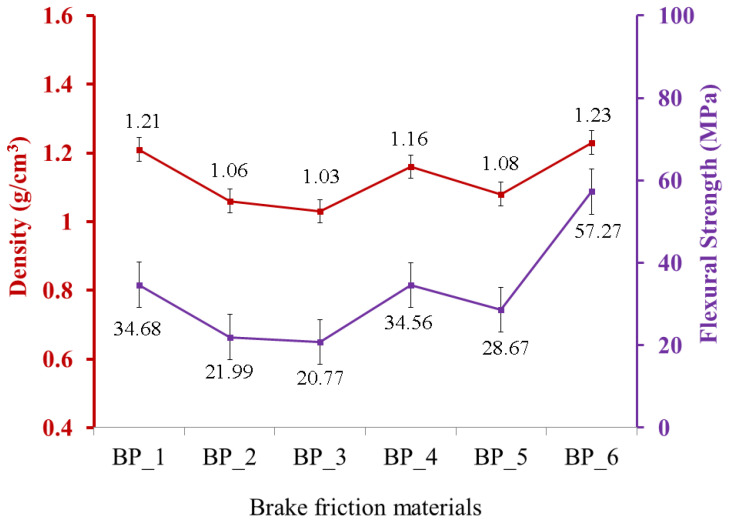
Relationship between density and flexural strength in brake friction materials.

Furthermore, the flexural strength produced in this study has the same trend as the density of each BFM specimen. The relationship between density and flexural strength produced in this study is illustrated in Figure 3. The red and purple lines in Figure 3 show density and flexural strength, respectively. The higher the density value, the higher the flexural strength of the BFMs specimen, and conversely [76,77]. The highest flexural strength was discovered in the BP_6 specimen since it has the highest density. In contrast, the lowest flexural strength was discovered in the BP_3 specimen since it has the lowest density compared to the other specimens.

The findings in this study are consistent with the results of a study conducted by Wasilewski et al. Their research suggests that increasing the density from 1.93 g/cm^3^ to 2.35 g/cm^3^ increased flexural strength from 17.9 N/mm^2^ to 19.5 N/mm^2^ [78]. The same result was also discovered in the study of Jeyanthi et al., which indicates that increasing the density of specimen composites for automotive frontal beams resulted in higher flexural strength [79]. In this study, the BP_6 specimen produced the highest flexural strength compared to the other specimens, which was 57.2 MPa. Note that the flexural strength in the BP_6 specimen was higher than the flexural strength values produced in previous studies, which were 19.5 N/mm^2^ [78], 19 MPa [80], 35 MPa [81], 50 MPa [82], and 55.32 MPa [83].

**Figure 4:**
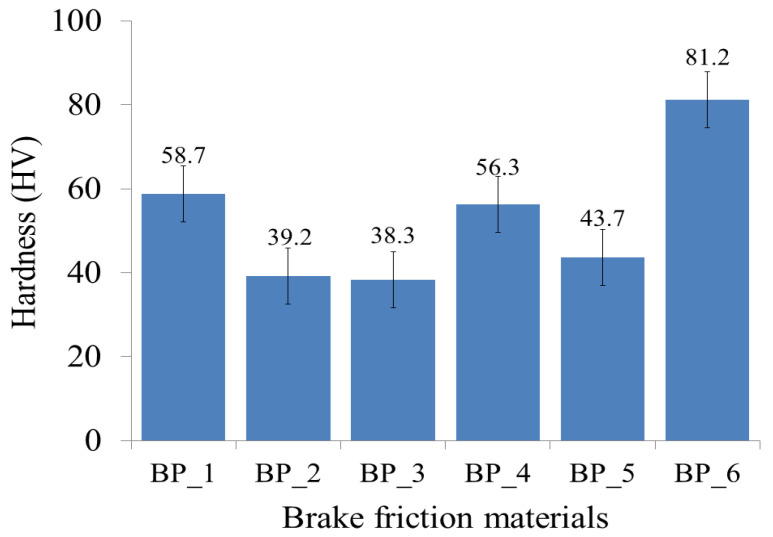
The hardness of brake friction materials.

The hardnesses of the BFMs with various compositions of the constituent materials are exhibited in Figure 4. The results of this study indicate that the addition of rice husk, Al_2_O_3_, and Fe_2_O_3_ greatly affects the hardness of the resulting BFMs. The BP_6 specimen produced a higher hardness than the other specimens, which was 81.2 HV. The results of this study suggest that the BP_3 specimen produced the lowest hardness, which was 38.3 HV; meanwhile, the hardness produced by the epoxy resin (specimen BP_1) is 58.7 HV. Based on the results, only the BP_6 specimen has a higher hardness compared to the hardness of the epoxy resin or BP_1 specimen.

Meanwhile, the BP_2, BP_3, BP_4, and BP_5 specimens have lower hardnesses compared to the hardness of the BP_1 specimen. This is due to the fact that the resulting densities for BP_2, BP_3, BP_4, and BP_5 are lower compared to the density of the BP_1 specimen.

**Figure 5:**
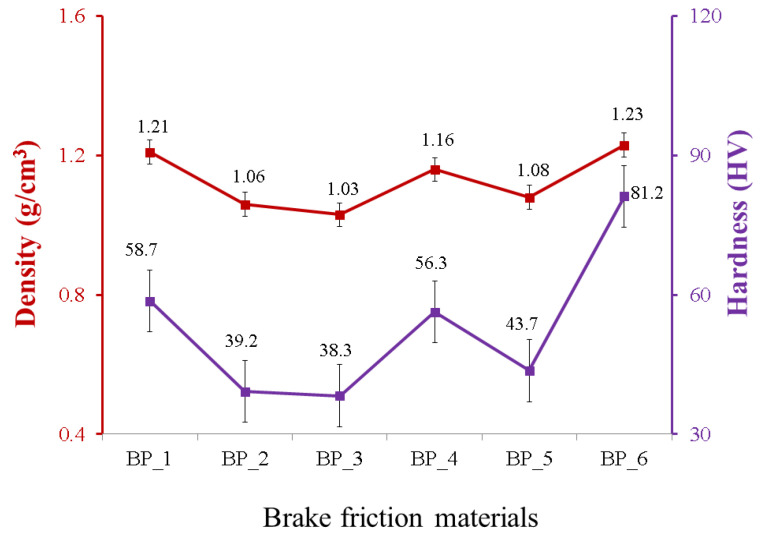
The relationship between the density and hardness of brake friction materials.

Furthermore, the hardness produced in this study has the same trend as the density of each BFM specimen. The relationship between density and hardness produced in this study is displayed in Figure 5. The red and purple lines in Figure 5 show density and hardness, respectively. The higher the density value, the higher the hardness of the BFM specimen, and conversely [26,76,84,85,86,87]. Note that the highest hardness was discovered in the BP_6 specimen, given that it has the highest density, while the lowest hardness was discovered in the BP_3 specimen, considering it has the lowest density compared to the other specimens. Incorporating rice husk, Al_2_O_3_, and Fe_2_O_3_ in epoxy resin increases the matrix’s resistance to indentations and plastic deformation, thereby increasing the hardness of the BP_6 specimen [84,87].

The findings in this study are consistent with the results of research conducted by Kasim et al. Their research suggests that an increase in pineapple leaf fiber (PLF) loading increased the density and hardness of the composite material [87]. Similar results were also established in a study by Shivakumar et al. [76] and Simsek et al. [84]. According to the study by Simsek et al., using hard oxide particles such as Al_2_O_3_ increases the density and hardness of a relatively ductile matrix in a linear trend [84]. Therefore, the BP_6 specimen produced a higher hardness compared to the results of the study conducted by Kholil et al. Their research produced brake linings from composites made from coconut coir, sawdust, and cow bone with a highest hardness of 35.4 HV [23]. However, the hardness of the BP_6 specimen was lower when compared to the research conducted by Abutu et al. and Daud et al., with their resulting BFMs having hardnesses of 63.31 (Shore D scale) [53] and 69.7 (Shore D scale) [19].

This study only performed SEM, wear, and TGA/DSC tests on the BP_1 and BP_6 specimens. This is because the BP_6 specimen produced higher values for the properties of (density, flexural strength, flexural modulus, and hardness) than the BP_1 specimen (control specimen). Meanwhile, the BP_2, BP_3, BP_4, and BP_5 specimens produced lower properties than the BP_1 specimen (control specimen). The results of the wear tests on the BP_1 and BP_6 specimens are provided in Table 4. This study shows that adding rice husk, Al_2_O_3_, and Fe_2_O_3_ greatly affects the specific wear rates of the resulting BFMs. The BP_6 specimen produced lower specific wear rates than the specific wear rates of the BP_1 specimen composed of 100 wt.% epoxy resin. Here, the specific wear rates of specimens BP_1 and BP_6 were 9.1 × 10^−7^ mm^2^/kg and 8.67 × 10^−7^ mm^2^/kg. The results of this study indicate that the BP_6 specimen has an advantage in terms of wear resistance compared to the BP_1 specimen. The greater the specific wear rate, the lower the wear resistance.

As previously explained, the combination of epoxy, rice husk, Al_2_O_3_, and Fe_2_O_3_ in the BP_6 specimen produced the highest density and hardness of 1.23 g/cm^3^ and 81.2 HV, respectively. Mousavi et al. stated that the higher the hardness, the lower the material’s wear rate [88]. The correlations of density, hardness, and specific wear rates for BFMs are listed in Table 4. The higher the hardness of the material, the more the material is resistant to scratches and penetration and the lower the specific wear rates. Note that the specific wear rate is a measure used to calculate how much material is worn or removed from the surface of a material in a certain period and under certain conditions, such as frictional force or pressure [89]. A smaller specific wear rate, meaning that less material is worn or removed from the material’s surface in the same amount of time, indicates that the material is more wear-resistant. Therefore, the lower the specific wear rate, the higher the material’s wear resistance [90,91,92].

Research by Liao et al. indicates that increasing the hardness of the material results in a decrease in the wear rate. The lower the specific wear value, the better the wear resistance properties of a material [93]. The findings of research conducted by Fitriyana et al. concur [94,95]. Hence, this study’s findings are coherent with the findings reported by Abutu et al. that concluded that rice husk could be employed effectively in brake pad formulations when combined with the appropriate additives. Utilizing rice husk significantly enhanced the overall performance of the BFM. A higher friction coefficient and lower wear rate can be obtained from brake pads made from the 20% rice husk sample [96]. Similarly, Acharya et al. investigated the effect of using rice husk to improve the tribological properties of epoxy composites. Their research suggests that the wear rate decreases as the concentration of rice husk fibers increases (from 5% to 10%). They discovered that 10% rice husk specimens exhibited the lowest wear rate. However, the wear rate of the composite increases when the rice husk content is greater than 10% [97]. According to Crǎciun et al., higher coconut fiber content improved frictional performance in a pin-on-disc test. During wear testing with the pin-on-disc method, specimens with a higher coconut fiber content (C3 and C4) produced a more stable friction coefficient compared with other specimens [67].

The specific wear rates produced by the BP_6 specimen (8.67 × 10^−7^ mm^2^/kg) are smaller than the specific wear rates produced in previous studies with specific wear rates of 6.9 × 10^−6^ mm^2^/kg [98], 3.3 × 10^−6^ mm^2^/kg [91], and 2.02 × 10^−6^ mm^2^/kg [91]. Nevertheless, the specific wear rates produced by the BP_6 specimen are greater than those of commercial products. The specific wear rates of commercial products are 6.5 × 10^−7^ mm^2^/kg and 5.9 × 10^−7^ mm^2^/kg, obtained during wear tests in dry and wet conditions [91].

**Figure 6:**
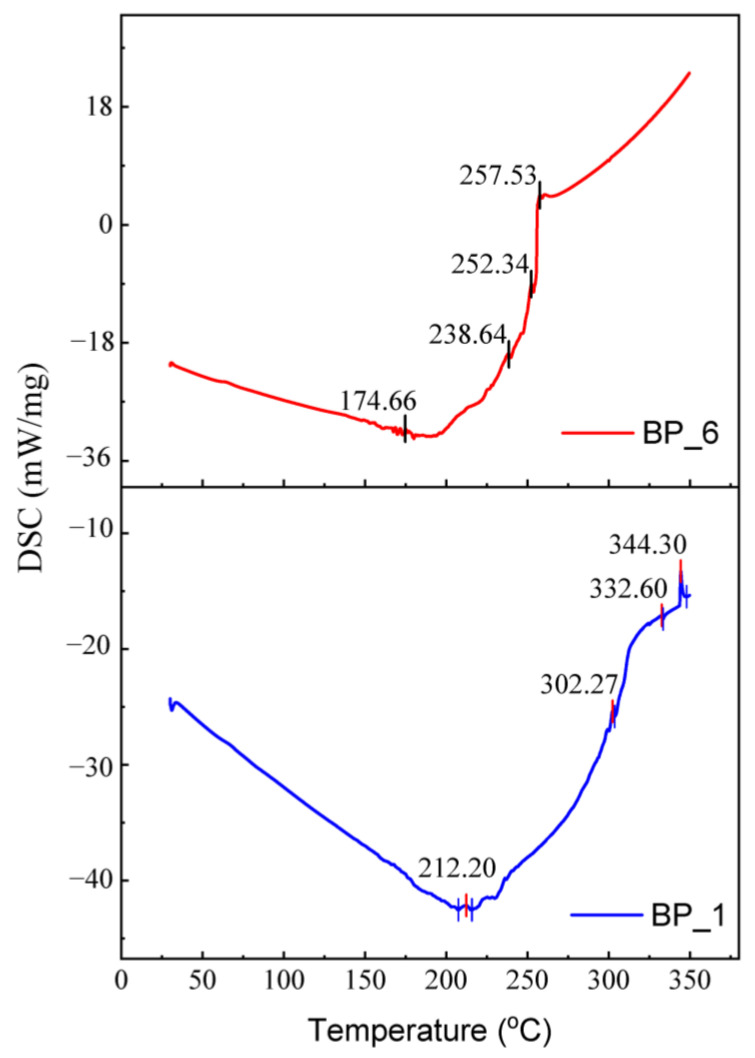
DSC test results of BP_1 and BP_6 specimens.

The results of the DSC test for specimens BP_1 and BP_6 are demonstrated in Figure 6. In this test, the specimens were analyzed at a heating rate of 20 °C/min in a temperature range of 30–350 °C. In the BP_6 specimen, a peak was established at 30–180 °C, indicating the presence of water molecules [52,99,100]. The presence of water molecules in the BP_6 specimen is indicated by the presence of a peak at 174.66 °C. No exothermic or endothermic reactions were observed in the 50 to 170 °C range, indicating that the specimens BP_1 and BP_6 were stable in this temperature range. Note that the thermal decomposition of hemicellulose starts at around 180 °C and ends at around 350 °C [52,100,101]. From the DSC test results graph, the decomposition of hemicellulose in BP_6 occurs at a temperature of 257.53 °C.

**Figure 7:**
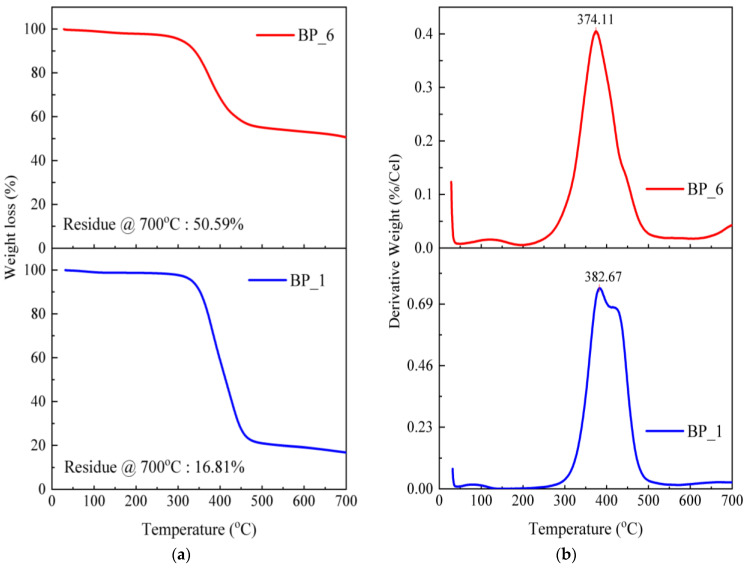
TGA (**a**) and DTG (**b**) curves of brake friction materials.

TGA testing applies heat to evaluate the thermal stability (strength of a material at a certain temperature), oxidative stability (rate of absorption of oxygen in a material), and compositional properties (e.g., fillers, polymer resins, solvents) of a sample with a set time and temperature [52]. In this study, the thermal decomposition of BFM composites occurred between 30 and 700 °C. The BP_1 specimen with a volume fraction of 100% epoxy resin experienced greater weight loss than the BP_6 specimen (Figure 7a).

The incorporation of rice husk, Al_2_O_3_, and Fe_2_O_3_ into the epoxy resin affects weight loss during the thermal degradation process. At 30–250 °C, the BF_6 specimen lost 2.63% of its initial weight. Meanwhile, the BP_1 specimen decreased by 1.5% from the initial weight at the same temperature range. The reduction in weight of the BP_6 specimen was greater than that of BP_1 in a temperature range of 30–250 °C since the hemicellulose in rice husks can absorb more moisture. During the thermal degradation process, evaporation of water vapor occurs; hence, the weight loss in the BP_6 specimen increased.

In the temperature range of 250–550 °C, the weight loss for specimen BP_6 is lesser than for specimen BP_1. Here, the weight loss in the BP_6 and BP_1 specimens was 43.37% and 78.7% of the initial weight. The results of this study indicate that incorporating rice husk, Al_2_O_3_, and Fe_2_O_3_ into the epoxy resin can reduce the decomposition temperature and increase the thermal stability of BFMs. This can be observed from the weight loss in the temperature range of 250–550 °C and the amount of residue produced when heated to 700 °C. The residues produced in the BP_6 and BP_1 specimens at the setting temperature of decomposition at 700 °C were 50.59% and 16.81%.

According to the findings, adding rice husk, Al_2_O_3_, and Fe_2_O_3_ to specimen BP_6 caused thermal stability and improved the initial and final decomposition temperatures. Other than that, adding rice husk reinforcement to composites with epoxy binders can reduce the decomposition temperature and increase thermal stability [102]. 

Figure 7b compares the DTG curves of the BP_1 and BP_6 specimens. In the BP_6 specimen, a small shoulder peak was found before (200–300 °C) and after the main peak or maximum decomposition temperature (T_max_) (about 500 °C). Typically, natural fiber composites have these shoulder peaks. While lignin decomposition can be linked to temperatures around 500 °C, the shoulders between 200 °C and 300 °C suggest the presence of hemicellulose. At a temperature between 350 and 400 °C, the epoxy molecular chains break and depolymerize, which causes the cellulose in natural fibers to decompose [100]. The main peaks on the DTG curve indicate T_max_ values occurring at 382.67 °C for the BP_1 specimen and 374.11 °C for the BP_6 specimen.

The results of this study indicate that the T_max_ of BP_1 (epoxy, 100 wt.%) was slightly higher than that of the BP_6 specimen. Consequently, the same results were obtained in previous studies [52]. This happens due to the higher thermal stability of the epoxy matrix, which is employed as a protective layer for the reinforcing fibers in BFMs. T_max_ is the degradation temperature, which is associated with the maximum weight loss. In addition, it is also an important indicator that indicates the material’s thermal stability [103]. This study’s results suggest a main peak or T_max_ in the DTG curve, which is almost the same between fiber and matrix. Note that the T_max_ of the BP_6 specimen is higher than the results of a study by Bashir et al. The results of their research obtained banana-fiber-reinforced brake lining composites with a T_max_ of 250 °C [104].

**Figure 8:**
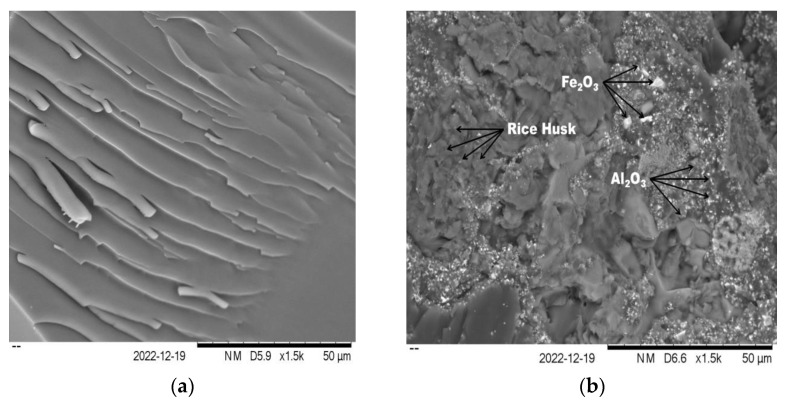
SEM images of the (**a**) BP_1 and (**b**) BP_6 specimens.

In order to qualitatively evaluate the epoxy, rice husk, Al_2_O_3_, and Fe_2_O_3_ interfaces, SEM analyses of the fracture surfaces of BP_1 and BP_6 specimens were conducted. A significant difference exists between the fracture surfaces of the BP_1 and BP_6 specimens (Figure 8). Figure 8 illustrates the SEM images of the fracture surface of the epoxy resin as a matrix (Specimen BP_1) and specimen BP_6. The SEM image of the epoxy resin (BP_1 specimens) suggests a smooth cleavage surface and barely visible thin cracks (Figure 8a). This is a distinctive characteristic of brittle failure and crack propagation via indirect paths. On the other hand, images obtained via SEM tests of the BP_6 specimen reveal the eventual presence of rice husk, Al_2_O_3_, and Fe_2_O_3_ particulate aggregates in the epoxy resin.

The absence of voids around the rice husk, Al_2_O_3_, and Fe_2_O_3_ particles in BP_6 specimens demonstrates good adhesion to the epoxy matrix. Moreover, the SEM image of the fracture surface of the BP_6 specimen indicates irregular cleavage due to the presence of rice husk, Al_2_O_3_, and Fe_2_O_3_ in the epoxy resin. This phenomenon is caused by a crack deflection mechanism when the crack propagates in the matrix and encounters rice husk, Al_2_O_3_, and Fe_2_O_3_ particles. This results in an increase in the stress intensity factor at the tip of the crack. This means that rice husk, Al_2_O_3_, and Fe_2_O_3_ are strong barriers against crack propagation. Therefore, the energy absorption capacity of the BP_6 specimen is higher than that of the epoxy resin (BP_1 specimen). The good interfacial adhesion between the epoxy matrix, rice husk, Al_2_O_3_, and Fe_2_O_3_ plays a significant role in improving the mechanical properties of the composite specimens. Note that the good interfacial adhesion between the matrix and reinforcement materials improves the mechanical properties of the composite specimens [105,106,107]. The results of this investigation are identical to those that Azizi et al. obtained. Due to the presence of nano zirconia in the matrix, the SEM image of the composite specimen’s fracture surface reveals irregular cleavage.

According to their research, the most effective method for inhibiting fracture propagation in composites was the addition of 3% zirconia. This causes the composite with 3% zirconia to have superior mechanical properties compared to epoxy resin [107].

## 4. Conclusions

BFM was successfully prepared using the hand layup method with varying concentrations of rice husk, Al_2_O_3_, and Fe_2_O_3_, which were incorporated into the epoxy resin. The results of this study indicate that the incorporation of rice husk, Al_2_O_3_, and Fe_2_O_3_ into epoxy resin has a significant effect on the resulting BFM properties such as hardness, density, flexural strength, flexural modulus, specific wear rate, and thermal stability. SEM images of the BP_6 specimen reveal the eventual presence of rice husk, Al_2_O_3_, and Fe_2_O_3_ particulate aggregates in the epoxy resin. Note that the absence of voids in the BP_6 specimen demonstrates good interfacial adhesion between the epoxy matrix, rice husk, Al_2_O_3_, and Fe_2_O_3_. The good interfacial adhesion between the matrix and reinforcement materials improves the mechanical properties of the composite specimens. This causes the BP_6 specimen to have better density, hardness, density, flexural strength, flexural modulus, specific wear rate, and thermal stability compared to other specimens. The BP_6 specimen has density, hardness, flexural strength, flexural modulus, and specific wear rates of 1.23 g/cm^3^, 81.2 HV, 57.24 MPa, 4.08 GPa, 8.665 × 10^−7^ mm^2^/kg, respectively.

According to the findings of this study, the use of Al_2_O_3_ and Fe_2_O_3_ powders with a concentration of 15 wt.% can produce effective interfacial adhesion, increasing the density of the BFM. The specimen’s hardness, flexural strength, flexural modulus, and thermal stability increase as the specimen’s density rises. However, employing Al_2_O_3_ and Fe_2_O_3_ in concentrations greater than 15 wt.% makes it challenging to acquire a homogeneous mixture, resulting in the formation of agglomerates and stress concentrations in the matrix. This can lead to an increase in voids, a decrease in interfacial bonding, and a decrease in density and other properties. Moreover, this is clearly observed in the BP_2, BP_3, BP_4, and BP_5 specimens, which utilized Al_2_O_3_ and Fe_2_O_3_ with a concentration exceeding 15 wt.%. The density, hardness, flexural strength, and flexural modulus values of the BP_2, BP_3, BP_4, and BP_5 specimens were lower compared to specimen BP_1, which applied 100% epoxy resin.

Furthermore, this study discovered that the BP_6 specimen had better specific wear rates and thermal properties than the BP_1 specimen, indicating that the combination of each component at a certain concentration increased the wear resistance and thermal stability of the BFM. These findings provide valuable insights into developing environmentally friendly and sustainable BFMs with suitable performance and durability for automotive applications with rice husk, Al_2_O_3_, and Fe_2_O_3_.

## Figures and Tables

**Table 1:** The properties of the Al_2_O_3_ and Fe_2_O_3_.

Specifications	Al_2_O_3_ [42]	Fe_2_O_3_ [43]
Boiling point (°C)	2980	3414
Density (g/cm^3^)	3.94 (at 20 °C)	5.25 (at 25 °C)
Melting point (°C)	2040	1565
Molecular weight (g/mol)	101.96	159.69

**Table 2:** The properties of the epoxy resin EPR 174 [44,45,46].

Specifications	Standard
Epoxy resin type	Bisphenol A-Epichlorohydrin
Hardener type	Cyclonliphatic Amine (EPH-555)
Density at 25 ℃ (g/cm³)	1.16 ± 0.02
Flexural strength (MPa)	81.3
Compressive strength (MPa)	88.2
Tensile strength (MPa)	63.7
Elongation (%)	6
Viscocity at 25 ℃ (mPa.s)	13,000 ± 2000
Flash point (℃)	>250

**Table 3:** Code and specimen composition of brake friction materials.

Specimen Code	Epoxy (wt.%)	Rice Husk (wt.%)	Al_2_O_3_ (wt.%)	Fe_2_O_3_ (wt.%)
BP_1	100	0	0	0
BP_2	50	0	25	25
BP_3	50	5	22.5	22.5
BP_4	50	10	20	20
BP_5	50	15	17.5	17.5
BP_6	50	20	15	15

**Table 4:** Comparison of density, hardness, and specific wear rates of BP_1 and BP_6 specimens.

Specimen Code	Density (g/cm^3^)	Hardness (HV)	Specific Wear Rates (10^−7^ mm^2^/kg)
BP_1	1.21	58.7	9.1
BP_6	1.23	81.2	8.67

## Data Availability

Data are contained within the article.
